# Competitive Inhibitory Effect of Calcium Polypeptides on Cd Enrichment of *Brassia campestris* L.

**DOI:** 10.3390/ijerph16224472

**Published:** 2019-11-14

**Authors:** Hongbing Chen, Fangfang Shu, Sheng Yang, Yadong Li, Shilin Wang

**Affiliations:** 1 Key Laboratory of Regional Development and Environmental Response of Hubei Province, Hubei University, Wuhan 430062 China; hbchen7112@163.com; 2 College of life Science, Hubei University, Wuhan 430062, China; fangfangshu123@163.com (F.S.); yangsheng1980@126.com (S.Y.); shilinwang201213@163.com (S.W.)

**Keywords:** calcium polypeptides, *Brassia campestris* L., calcium, cadmium, competitive inhibition

## Abstract

Most cadmium-polluted farmland and land surrounding mining areas are difficult to repair and control, seriously threatening the food safety of the crops planted in these regions. As an essential element for plant growth, calcium plays an important role in stress-resistance regulation. In this study, *Brassia campestris* L. was used as the experimental material and polluted soil with cadmium was used as the experimental soil sample, to explore the competition inhibition of calcium polypeptide application on the absorption of Cd^2+^ by *Brassia campestris* L. in the growth process, as well as the effect of calcium application on the growth. Results showed that the application of calcium polypeptides significantly promoted the growth of *Brassia campestris* L. Calcium polypeptides could be used as high-quality fertilizer, alleviating the effect of Cd^2+^ stress on the growth of *Brassia campestris* L., and promoting the absorption of K^+^, Ca^2+^, and other nutrients by *Brassia campestris* L. Under different calcium polypeptide application conditions, the effective state of Cd^2+^ in the soil showed less significant difference, indicating that the calcium polypeptide had weak or limited passivation effects on Cd^2+^. There was a significantly negative correlation between Cd concentration in *Brassia campestris* L. and calcium application (*r* = −0.99, *p* < 0.01) when calcium polypeptide was over-applied, which indicates that the inhibition effect of Cd^2+^ absorption on *Brassia campestris* L. is mainly through competitive inhibition rather than passivation. The results showed that calcium polypeptide has dual functions on the competitive inhibition of heavy metals and a good fertilizer effect, providing a new technology for in situ remediation of heavy-metal pollution, and a new approach for the treatment of cadmium-contaminated farmland and surrounding mining land.

## 1. Introduction

Cadmium (Cd^2+^) is a highly toxic heavy-metal element. Due to unreasonable sewage irrigation, the diffusion and deposition of industrial dust, agricultural sludge, and the use of cadmium-containing phosphate rock powder, increasing amounts of cadmium have entered farmland [1,2,3,4]. In recent years, soil pollution from heavy metals has become increasingly serious, especially in farming soil. According to incomplete statistics, the area of cultivated land polluted by heavy metals in China is about 10 million hectares, accounting for more than 8% of the total cultivated-land area. According to the National Soil Pollution Survey Bulletin issued in 2014, inorganic pollution was the main type, for which Cd exceeded the standard by 7%, which was much higher than that for other heavy metals. Moreover, most cadmium-polluted agricultural land and regions surrounding mining areas in China are still planting bases with large areas and are very difficult to remediate, which seriously threatens the food safety of crops planted in these regions. The amount of agricultural products produced each year that exceed the cadmium standard has reached 1.46 billion kg [5]. Due to its strong biological migration, cadmium is easily absorbed and accumulated by plants, which seriously affects crop yield and quality. Moreover, cadmium enters the human body through the food chain, i.e., soil–plant–(animal)–human, which can cause a series of diseases in the human body and greatly harm people’s health [6,7,8].

Cadmium is an indispensable element [6] that has been proven to be harmful to plant growth and development, such as by inhibiting water uptake and transport [7]; interfering with the absorption, transportation, and use of Ca, Mg, P and K; inhibiting nitrate reductase activity, reducing nitrate uptake and transport, and inhibiting photosynthesis and respiration [9,10]; and causing disorders in energy conversion and protein synthesis, leading to nutritional imbalance and ultrastructural damage [11,12]. High cadmium accumulation can also cause heavy-metal-poisoning symptoms and reduce plant biomass [13,14,15]. Plants respond to Cd^2+^ stress through different physiological and biochemical mechanisms [16,17]. In view of this, increasing attention has been paid to how to relieve or alleviate Cd^2+^ stress [3,4,18,19].

Calcium is one of the most abundant elements in soil, and it is also an essential nutrient element in the process of plant growth and development, which has extremely important physiological functions. For many years, research on the function of calcium has been a very active field, and its role in plant adversity has become increasingly clear. Calcium plays an important role in the selective absorption of ions, growth, senescence, information transmission, and the stress resistance of plants as a link between phosphoric acid and protein carboxyl in cell membranes. It plays a central regulatory role in plant growth and development, and in the response to environmental stress [12]. It is recognized as the central regulator of plant biochemical and physiological processes that help to reduce the toxicity of heavy metals to plants [20]. Because the ion radius of Ca^2+^ is close to that of Cd^2+^ and the charge is the same, Ca^2+^ and Cd^2+^ have similar physical and chemical properties. Calcium is the main competitor of cadmium-adsorption sites in soil. When calcium and cadmium coexist, the two interact, calcium can reduce the content of available cadmium in soil, and the degree of cadmium stress in plants is weakened [12,21,22]. At the same time, it is an important way for plants to absorb cadmium through the Ca^2+^ channel. Calcium can also reduce stress by regulating physiological or metabolic changes induced by cadmium [23]. Many scholars believe that calcium (Ca^2+^) can be used as a secondary messenger to combine with calmodulin (CaM) to couple extracellular signaling and intracellular physiological and biochemical reactions, as well as improve the resistance of plants to adversity by stabilizing cell-wall and -membrane structure [24] and by inducing the expression of specific genes. It is also believed that cadmium and calcium compete for transporters on plant plasma membranes that enter root cells in the form of ions or metal chelates and are absorbed by plants. Therefore, excessive application of calcium reduces the amount of cadmium that enters plants. In recent years, studies have shown that the exogenous application of calcium (Ca^2+^) can reduce cadmium toxicity in *Arabidopsis*, tobacco, and maize seedlings [25]. Ca^2+^ can participate in many kinds of life activities and the anti-adversity metabolism process of plants. Exogenous Ca^2+^ can enhance plant adaptability to many types of abiotic adversity, improve their tolerance to heavy-metal toxicity to reduce the damage caused to a certain extent, reduce plant absorption of Cd^2+^ and the damage that cadmium stress causes, and improve the yield and quality of crops. The effect of Ca^2+^ on the accumulation and transportation of Cd^2+^ is dose-dependent [26]. When the amount of calcium is 2.0–3.0 mmol·L^−1^, the effect of Ca^2+^ on the absorption and transport of Cd^2+^ in plants is the most significant. When the amount of Ca^2+^ is 5.0 mmol·L^−1^, it reaches the peak. It was found that 5.0 mmol·L^−1^ Ca^2+^ significantly reduced the accumulation of Cd^2+^ in alfalfa [26]. On the contrary, under the treatment of 1.0 mmol·L^−1^ Ca^2+^, the absorption and transport of Cd^2+^ in plants were enhanced. However, excessive application of Ca^2+^ also inhibited plant growth [27]. Some scholars studied the effect of calcium on the Cd^2+^ toxicity of *Arabidopsis* seedlings and found that Ca^2+^ supplementation restored normal auxin transport in plants, thus alleviating cadmium-induced plant-growth inhibition [26,27,28]. The addition of exogenous Ca^2+^ also helps to enhance the absorption of basic mineral elements that are conducive to plant growth and development. For example, 0.1 mmol·L^−1^ Cd^2+^ significantly reduced the root, axis, and hypocotyl length of maize seedlings. The application of 10 mmol·L^−1^ Ca^2+^ offset the harmful effects of cadmium on maize growth [29,30].

At present, fixed in situ remediation technology has good application prospects in the treatment of heavy-metal pollution in soil due to its characteristics, i.e., a short remediation time, high efficiency, less investment, and easy to operate [20,31,32,33]. Lime, organic fertilizer and inorganic calcium salt (such as calcium chloride and calcium nitrate), phosphates (such as calcium magnesium phosphate, superphosphate), and other soil improvers are the most commonly used and effective types of heavy-metal soil-pollution-treatment technology so far. Chinese farmers have long used calcium-containing materials such as lime, gypsum, bone powder, and plant ash as fertilizers; some farmers in areas of South China are still in the habit of using them. The production of some areas increased with the use of calcium-containing fertilizers applied to crops. A soil-remediation agent with a high calcium content is needed, that not only has a fertilizer effect but that can also promote plant growth and mitigate heavy-metal pollution. 

The disadvantage of phosphate compounds is that water eutrophication caused by phosphate loss is extremely serious, so it has not been widely used [34,35]; the application of lime materials can also easily cause problems such as soil hardening and changes in physical and chemical soil properties [36]; inorganic salt fertilizers such as calcium nitrate and calcium chloride can rapidly increase the Ca^2+^ content in soil and can also provide nitrogen for plant growth, but it can cause soil acidification with long-term application and is not suitable for large-scale use [37]. Common organic passivators have a good control effect on cadmium-polluted soil, but there are also problems such as low passivation efficiency, slow passivation speed, ease of causing nonpoint source pollution, which seriously affects surrounding water sources, and the effect of organic fertilizer on soil pH that gradually disappears with the decomposition of organic matter.

On the basis of this, our research team developed an in-situ peptide calcium salt repair agent (calcium polypeptide) [38,39,40] that is an organic metal chelate with a ring structure generated by the chelation reaction between peptide and Ca^2+^; it is also a new protein organic fertilizer [41]. It is mainly hydrolyzed by adding quicklime to animal and plant waste proteins such as soybean meal, animal hair and skin, hoof nails, skeletons, and rhizobium under high temperatures. It is formed by combining a metal calcium ion with a peptide by a covalent bond according to a certain molar ratio. The average molecular weight is within 5000 Dalton, and the solution is alkaline, which allows important biological activity. Calcium polypeptide can improve the bioavailability of metal ions with the help of peptides and can facilitate the absorption of organic amino acid fertilizer by crops with good biocompatibility. It has good solubility and high nutrition. It has physiological characteristics with which other organic fertilizers cannot compare. Calcium polypeptide has low cost, a simple manufacturing process, and has many advantages that other organic fertilizers do not. It is regarded as a higher-value organic fertilizer [42,43]. Some studies have shown that protein organic fertilizers have the same effect as a urea fertilizer and can improve the microbial-community structure in the soil, increase plant chlorophyll content, and promote rapid plant growth [44,45]. Some studies also found that coordination between metal ions and peptides occurs in amino, imino, or carboxyl groups [46], and they are combined in the form of a single-tooth covalent bond [47,48]. The chelation reaction of calcium polypeptides is relatively complex, involving the coordination and combination of metal ions with peptide amino and carboxyl groups, as well as the separation of metal ions from peptide carboxyl groups [49]. In agricultural production, calcium polypeptides can prevent the side effects of calcium and other substances, promote the absorption and utilization of calcium by crops, and reduce the stress caused by heavy-metal ions [50,51]; therefore, calcium polypeptides have great potential in the remediation of heavy-metal-contaminated soil [52].

At present, there are many studies on Ca^2+^ regulating Cd^2+^ stress in plants [39,40,53], but there are few reports on the research of heavy-metal-contaminated-soil remediation agents that not only competitively inhibit heavy metals but also improve soil and crop quality. On the basis of this, we used *Brassia campestris* L. as an experimental material, using self-made calcium polypeptide to act in cadmium-bearing soil to explore the effects of different concentrations of calcium polypeptide on plant growth, crop quality, and the competitive inhibition effect on plant absorption of Cd^2+^, so as to provide a new way for the treatment of farmland and mining areas polluted with Cd^2+^, and to facilitate the cultivation of high-quality agricultural products.

## 2. Methods and Materials

### 2.1. Experimental Materials

#### 2.1.1. Test Plants

*Brassia campestris* L. is classified as *Brassica* of *Cruciferae*. It is an annually cultivated *Brassica* (mustard) plant with edible stems and leaves, a short growth period, rich mineral elements, and it is sensitive to heavy-metal pollution. *Brassia campestris* L. was selected as the experimental material. The experiment was carried out in the solar greenhouse of the College of Life Sciences, Hubei University. The greenhouse is located at N 3034′ 56.61, E 11419′ 40.48. *Brassia campestris* L. seeds were purchased from Tianjin Jinke Lifeng Seedling Co., Ltd. In this experiment, *Brassia campestris* L. seeds were sown in loose soil that was kept moist, and seedlings were transplanted into the test basin after the germination and growth of 2 true leaves.

#### 2.1.2. Experiment-Soil-Sample Preparation 

The tested soils were collected from the surrounding soils of Hubei University and were neutral red loam (detailed index parameters shown in Table 1). We naturally dried the soil, removed stones and animal and plant residues visible to the naked eye, crushed the soil through a 2.0 mm sieve, mixed it evenly, and stored it in plastic buckets. Calculated and formulated the required cadmium chloride solution according to the weight of the soil and sprayed evenly on the soil in a spraying basin. The total cadmium concentration reached 2.0 and 5.0 mg·kg^−1^. After even mixing, the solution was added to a plastic bucket with a lid. Deionized water was added to the bucket according to 80% of field water capacity with a lid and stabilized for 30 days. During stabilization of the cadmium soil, the lid was opened, ventilated, water was added, and keep moist every 3 days. After that, the soil was air-dried, sieved through a 2.0 mm mesh, and put in a cool place for storage [38].

#### 2.1.3. Calcium Polypeptide Preparation

Calcium polypeptide is a new kind of in situ competitive inhibitor made by mixing plant meal (containing 40–45% protein) with quicklime for 1–6 h at 130 °C; the added amount of quicklime is 10–45% (W/W) of protein waste. Calcium polypeptide is transformed from the protein components in vegetable meals under high temperatures. 

### 2.2. Experimental Design

In recent years, cadmium pollution of farmland soil in China has mainly been caused by waste-water irrigation, improper fertilization, and the mining of metal minerals. The Cd^2+^ content of most farmland soil is 0.3–2.0 mg·kg^−1^, and the Cd^2+^ content of surrounding soil in mining areas is 2.5–23.0 mg·kg^−1^. However, these polluted farmlands and surrounding land in mining areas are still planting bases, which are not only large in area, but are also very difficult to remediate and deal with. It seriously threatens the food safety of crops planted on it. Therefore, clean (noncontaminated) soil was selected as the control soil in this test, and polluted soil with cadmium concentrations of 2.0 and 5.0 mg·kg^−1^ was selected as the test soil, which represents the soil from most farmlands and the surrounding mining areas, respectively. Three parallel test groups were set up, marked as Cd 0, Cd 2, and Cd 5, respectively. In each parallel experimental group, 7 treatment groups (including an unfertilized blank control group) were set up, 2.0 kg of cadmium-polluted soil samples were weighed in each basin, and 6 different calcium application gradients (0, 0.42, 0.84, 1.26, 1.68, and 0.21 g·kg^−1^, calculated by Ca^2+^) were set up to apply calcium polypeptide, which provided exogenous calcium and some nitrogen according to the same nitrogen-application amount (the most suitable application for *Brassia campestris* L.) The amount of nitrogen was 0.33 g·kg^−1^, and urea was used to supplement the nitrogen application. *Brassia campestris* L. sprouted in loose soil, was allowed to grow to 2 real leaves, and then transplanted. Fresh weight, *Chlorophyll a*, Ca^2+^, and Cd^2+^ were measured 40 days after transplanting.

### 2.3. Sample Processing and Index Measurement

#### 2.3.1. Fresh-Weight Determination

*Brassia campestris* L. was first washed with tap water, then with distilled water 3 times, and then dried with absorbent paper, and its fresh weight was measured.

#### 2.3.2. *Chlorophyll a* Determination 

The *Chlorophyll a* of leaves in the same treatment of plants were measured with a *chlorophyll a* analyzer, and the average values were measured 3 times in parallel.

#### 2.3.3. Determination of Calcium, Nitrogen, Phosphorus, Potassium, and Cadmium Contents in *Brassia campestris* L.

*Brassia campestris* L. samples were washed with tap water, then rinsed with deionized water. After being dried at 105 °C for 20 min, *Brassia campestris* L. samples were dried to constant weight at 80 °C. The *Brassia campestris* L. samples were crushed with a stainless-steel mill (HX-200, Zhejiang Yongkang Xi’an medical device factory, Yongkang City, China) and sieved by 60 mesh sieves. The contents of calcium, nitrogen, phosphorus, and potassium in *Brassia campestris* L. were determined with reference to the national PRC standards for the “Determination of copper, iron, zinc, calcium, magnesium and phosphorus in vegetables and their products” (GB/T23375-2009) and the agricultural standard for the “Determination of nitrogen, phosphorus and potassium in plants” (NY/T21017-2011). According to the national food-safety standard “Determination of cadmium in food” (GB5009.15-2014), the cadmium content in *Brassia campestris* L. was determined by the dry-ashing method. According to the method, 0.3–0.5 g of the dry sample was weighed into a porcelain crucible, it was carbonized on an adjustable electric furnace (DB-2A, Tianjin Gongxing electric appliance factory, Tianjin City, China) with a small fire until it was smoke free, followed by a transfer into a muffle furnace (SX2-2.5-10, Hubei Yingshan Jianli electric furnace manufacturing Co., Ltd., Huanggang City, China) for ashing at 500 °C for 6–8 h and then cooling. We dissolved the ash with a 1% nitric acid solution, transferred the sample into a 10 mL volumetric flask, rinsed all utensils and transferred the washed water into the volumetric flask, and mixed it with a 1% nitric acid solution at a constant volume for standby, while carrying out the reagent blank test and then a machine test.

#### 2.3.4. Determination of Available Cadmium in Soil

We referred to “Determination of Effective Lead and Cadmium—Atomic Absorption Spectrometry” (GB/T 23739-2009) for the determination of available cadmium in soil. The specific method entails taking 5.00 g of air-dried soil sample that had been passed through a 2 mm sieve, putting it into a 100 mL conical flask with a stopper, adding 25.00 mL of diethyltriamine pentaacetic acid (DTPA), extracting the agent with a pipette, shaking it horizontally 180 times per min at room temperature (25 ± 2 °C), extracting it for 2 h, followed by centrifugation or dry filtration (5–6 mL of the initial filtrate was discarded), and then testing the filtrate on atomic absorption spectrophotometer (WYG-2200, Anhui Wanyi science and technology Co., Ltd, Hefei City, China).

### 2.4. Data-Processing Method

By using Microsoft Office Excel 2016 (Microsoft Corporation, Redmond, Washington, USA) and IBM SPSS 22.0 (International Business Machines Corporation, Armonk, NY, USA) statistical software, we analyzed the experimental data by one-way ANOVA and LSD. Mean-value analysis of the samples was tested with the *t*-test to determine whether there were significant differences between the test results of the processing indicators (expressed by different letters). Then, we performed a Pearson correlation analysis of the parameters.

## 3. Results

### 3.1. Effects of Calcium Polypeptide on Growth Characteristics of Brassia campestris L.

**Figure 1:**
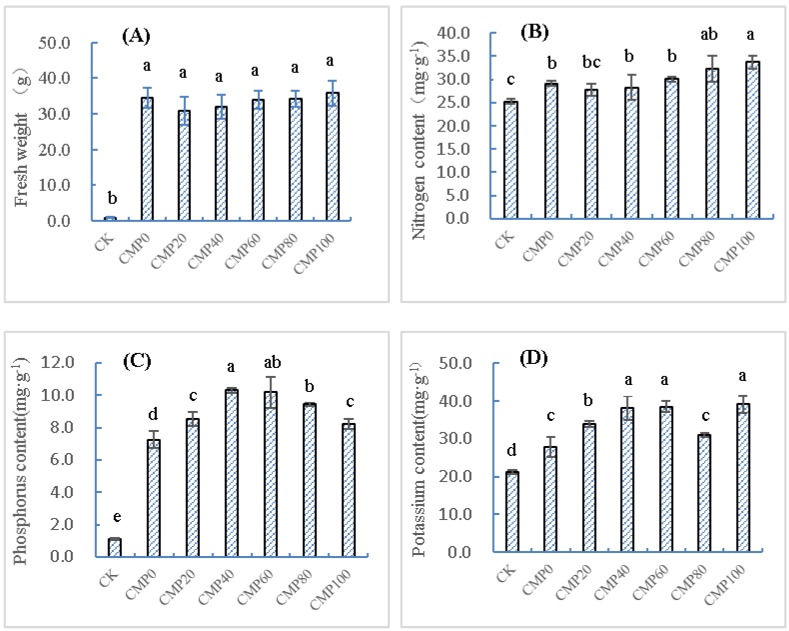
Changes in the fresh weight and contents of N, P, and K of *Brassia campestris* L. in raw soil: (**A**) Changes of fresh weight in *Brassia campestris* L. under different treatments; (**B**) Changes of Nitrogen content in *Brassia campestris* L. under different treatments; (**C**) Changes of Phosphorus content in *Brassia campestris* L. under different treatments; (**D**) Changes of Potassium content in *Brassia campestris* L. under different treatments. Different lowercase letters such as a, b, c, d, e, etc. represent significant differences (*p* < 0.05) between different treatment groups. CK stands for control group; CMP0, 20, 40, 60 and 100 stand for treatment groups of urea and calcium polypeptide, according to the same amount of nitrogen and different proportions of calcium application.

The combination of urea and calcium polypeptide was evenly mixed with normal soil according to the same amount of nitrogen and different proportions of calcium application. Sampling measurement was carried out 40 days after transplanting *Brassia campestris* L. seedlings. As shown in Figure 1A, the results showed that the growth trend of *Brassia campestris* L. was obvious in the fertilization treatment group. At 40 days, *Brassia campestris* L. seedlings were sampled and measured. The maximum fresh weight treatment for per plant was CMP100 (35.73 ± 3.54 g), and fresh weight of CMP0 treated with urea only was 34.40 ± 2.81 g. The fresh weight of CMP100 was 35.8 times higher than that of the control group (0.97 ± 0.11 g; *p* < 0.05); The treatment of CMP100 was 3.90% higher than that of the urea (CMP0), but there was no significant difference (*p* > 0.05). Results showed that calcium polypeptide could be used as a quick-acting fertilizer to rapidly promote plant growth, and its fertilizer effect was similar to that of the conventional urea (CMP0; *p* > 0.05).

As shown in Figure 1B, the N content of *Brassia campestris* L. was the highest in the CMP100 treatment at 40 days, which was 33.73 ± 1.46 mg·g^−1^ (dry weight, DW), 34.00% higher than that of CK, 19.00% higher than that of the CMP0 group, and the difference between CMP100 and CMP0 was significant (*p* < 0.05). The N content of *Brassia campestris* L. increased with the increase in the calcium polypeptide dosage in different proportions and showed a significant positive linear relationship (*r* = 0.87) between them.

As shown in Figure 1C, the P content of *Brassia campestris* L. in different treatments significantly increased at 40 days compared with the control group. The P content first increased and then decreased with the increase in the calcium application ratio. CMP40 had the best effect, reaching a peak value of 10.29 ± 0.14 mg·g^−1^ (DW). Based on these data (40 days of growth) of *Brassia campestris* L., the increase in the P content was consistent with the growth of *Brassia campestris* L. Compared with the blank test group, the P content in *Brassia campestris* L. was significantly different (*p* < 0.01). Within a certain range, P concentration increased with the increase in calcium application. However, after reaching a peak value, there was a significant downward trend, indicating that the application of appropriate calcium polypeptide could significantly improve the P absorption of *Brassia campestris* L., but excessive calcium would inhibit P absorption.

As shown in Figure 1D, CMP100 had the best effect on K^+^ uptake in *Brassia campestris* L. At 40 days, the content of K^+^ was 39.11 ± 2.26 mg·g^−1^ (DW), 80.49% higher than that of the control group, and 52.02% higher than that of CMP0. The K^+^ concentration in *Brassia campestris* L. increased with the increase in calcium polypeptide application. The K^+^ content in different treatments was significant difference (*p* < 0.01) from that of the blank test group (CK). 

### 3.2. Control of Brassia campestris L. Growth in Cadmium-Contaminated Soil by Different Calcium Polypeptide Application Amounts

**Figure 2:**
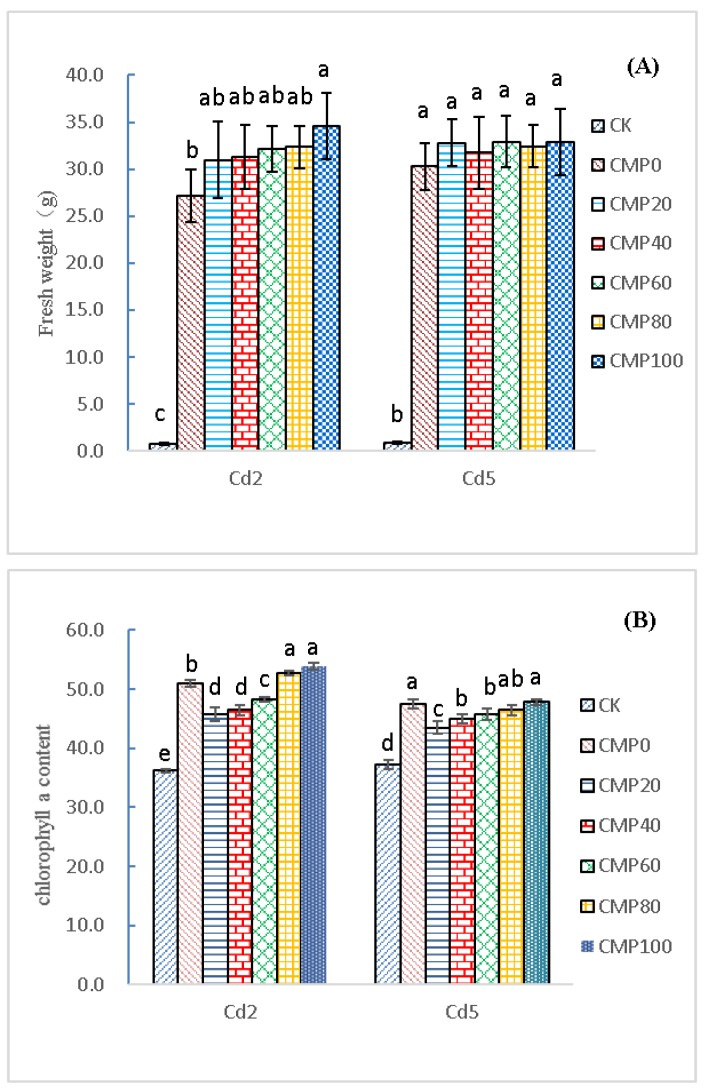
Effects of calcium application on the fresh weight and *Chlorophyll a* of *Brassia campestris* L.: (**A**) Changes of fresh weight in *Brassia campestris* L. under different treatments; (**B**) Changes of chlorophyll a content in *Brassia campestris* L. under different treatments. Different lowercase letters such as a, b, c, d, e, etc. represent significant differences (*p* < 0.05) between different treatment groups. Cd2 and Cd5 indicate that 2.0 and 5.0 mg·Kg^−1^ cadmium-contaminated soil samples were used as the test soil samples; CK stands for control group; CMP0, 20, 40, 60 and 100 stand for treatment groups of urea and calcium polypeptide, according to the same amount of nitrogen and different proportions of calcium application.

As shown in Figure 2A, *Brassia campestris* L. grew rapidly during the 40-day growth period. On the 40th day, the fresh weight of CK was 0.747 ± 0.12 g in the contaminated soil containing 2.0 mg·kg^−1^ cadmium. With the increase in calcium, the fresh weight of *Brassia campestris* L. gradually increased. The maximum fresh weight of *Brassia campestris* L. treatment group was CMP100 with the value of 34.63 ± 3.54 g. Compared with that of the CMP0 group (27.15 ± 2.46 g), CMP100 had 27.53% higher weight than CMP0 and showed a positive significant difference (*p* < 0.05). In the 5.0 mg·kg^−1^ cadmium-containing soil, the fresh weight of CK was 0.82 ± 0.04 g, and the maximum fresh weight treatment was CMP100 with the value of 32.89 ± 4.53 g. Compared with the CMP0 (30.26 ± 2.45 g), the value of CMP100 was 12.84% higher than that of CMP0 and showed a positive significant difference (*p* < 0.05). All the same, the value of CMP100 was 39.11 times higher than that of CMP0 and showed an extremely positive significant difference (*p* < 0.01).

As shown in Figure 2B, *Brassia campestris* L. was sampled after 40 days of growth. In the contaminated soil containing 2.0 mg·kg^−1^ cadmium, the *Chlorophyll a* content of different fertilization treatments was significantly different from that of CK (*p* < 0.05). The CMP100 treatment had the best effect on the *Chlorophyll a* content of *Brassia campestris* L., with a content of 53.86 ± 0.59; and the CK content of the control was 36.15 ± 0.20. The content of CMP0 was 50.93 ± 0.55, 5.72% (*p* > 0.05) higher than that of CMP0. 

In the soil contaminated with 5.0 mg·kg^−1^ of cadmium, *Chlorophyll a* was significantly different between different fertilization treatments and CK (*p* < 0.05). The CMP100 treatment had the best effect on the *chlorophyll a* content of *Brassia campestris* L., and its content was 47.78 ± 0.52, which was 0.61% (*p* > 0.05) higher than that of the CMP0 group. Compared with urea, the fresh weight of *Brassia campestris* L. under calcium polypeptide application was 1.28 times (*p* < 0.05) and 1.13 times (*p* < 0.01) higher than that with urea, and *chlorophyll a* was 5.72% (*p* > 0.05) and 0.61% (*p* > 0.05) higher than that with urea, in the soil contaminated with 2.0 mg·kg^−1^ and 5.0 mg·kg^−1^. 

The results showed that calcium polypeptide had a more obvious promoting effect on the growth of *Brassia campestris* L. than urea, which made better quality *Brassia campestris* L.

### 3.3. Effects of Calcium Polypeptide on Ca Content of Brassia campestris L.

**Figure 3:**
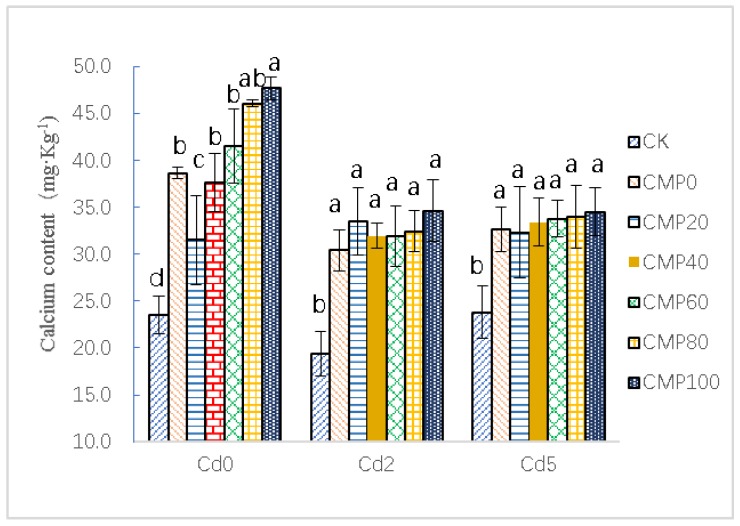
Effects of calcium application on the Ca content of *Brassia campestris* L. Cd0, Cd2, and Cd5 indicate that raw soil (noncontaminated) and 2.0 and 5.0 mg·Kg^−1^ cadmium-contaminated soil samples were used as the test group; CK stands for control group; CMP0, 20, 40, 60 and 100 stand for treatment groups of urea and calcium polypeptide, according to the same amount of nitrogen and different proportions of calcium polypeptide application. Different lowercase letters such as a, b, c, d, etc. represent significant differences (*p* < 0.05) between different treatment groups.

As shown in Figure 3, Ca^2+^ enrichment of *Brassia campestris* L. in raw soil increased with the increase of Ca application. The CMP100 treatment had the best Ca^2+^ enrichment effect on *Brassia campestris* L. The maximum Ca^2+^ enrichment group was CMP100 with the value of 47.67 ± 1.23 mg·g^−1^ (DW), 38.45% higher than that of CMP0, and showed a positive significant difference (*p* < 0.01). Besides CMP0, Ca^2+^ enrichment increased with the increase in calcium application and showed a positive significant linear relationship (*r* = 0.983 > 0.8). At 40 days, there were significant differences in the contaminated soil containing 2.0 mg·kg^−1^ of cadmium between different treatments and CK (*p* < 0.01). The CMP100 treatment had the best effect on Ca^2+^ enrichment in *Brassia campestris* L., with the highest concentration of 34.61 ± 3.28 mg·g^−1^ (DW). The content of Ca^2+^ in the CK group was 19.40 ± 2.37 mg·g^−1^ (DW), 78.42% higher than that of the CK group in the CMP100 group, and 56.68% higher than that of the CK group with a CMP0 concentration of 30.40 ± 2.20 mg·g^−1^ (DW). Among the contaminated soils with 5.0 mg·kg^−1^ of cadmium, there were significant differences between different fertilization treatments and CK. The CMP100 treatment had the best effect on the Ca^2+^ enrichment in *Brassia campestris* L., with a maximum of 34.52 ± 2.56 mg·g^−1^ (DW). The content of Ca^2+^ in CK group was 23.82 ± 2.84 mg·g^−1^ (DW), 44.90% higher in the CMP100 group than it was in the CK group, 37.07% higher in the CMP0 group (32.65 ± 2.32 mg·g^−1^ DW) than it was in the CK group, and 35.70% to 44.90% higher in the conventional nitrogen application CMP group than it was in the CK group (*p* < 0.01). Therefore, direct application of calcium polypeptide in cadmium-contaminated soil with conventional nitrogen application can significantly improve the calcium accumulation of *Brassia campestris* L.

### 3.4. Effects of Calcium Polypeptide on Cd^2+^ Content of Brassia campestris L.

**Figure 4:**
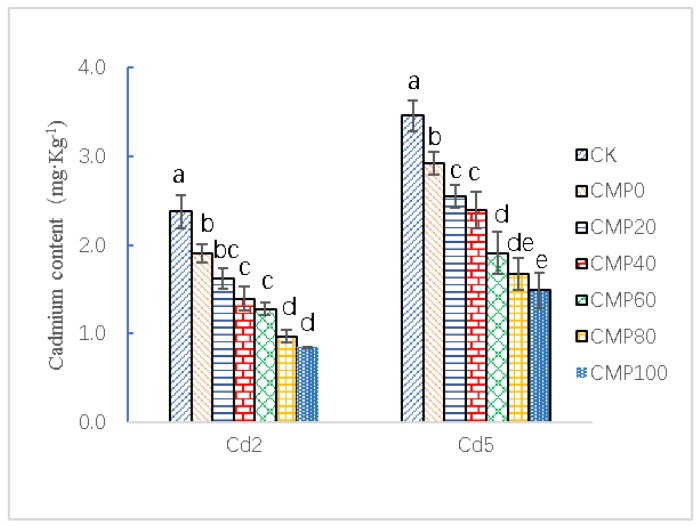
Effects of calcium application on the Cd content in *Brassia campestris* L. Cd2 and Cd5 indicate that 2.0 and 5.0 mg·Kg^−1^ cadmium-contaminated soil samples were used as the test group; CK stands for control group; CMP0, 20, 40, 60 and 100 stand for treatment groups of urea and calcium polypeptide, according to the same amount of nitrogen and different proportions of calcium application. Different lowercase letters such as a, b, c, d, e, etc. represent significant differences (*p* < 0.05) between different treatment groups.

As shown in Figure 4, soil contaminated with 2.0 mg·kg^−1^ of cadmium (CMP100 treatment) had the best effect on reducing Cd^2+^ enrichment in *Brassia campestris* L. compared with the CK treatment at 40 days. The lowest Cd^2+^ enrichment was 0.84 ± 0.003 mg·kg^−1^. The CMP100 treatment was 64.71% lower than that of the CK control, and conventional calcium treatment was more effective than that of the CK control. There was a significant difference between the two groups (*p* < 0.01). It can be concluded that the Cd enrichment of *Brassia campestris* L. gradually decreased by increasing the application of calcium polypeptide. Similarly, in the contaminated soil containing 5.0 mg·kg^−1^ of cadmium, CMP100 was the best among the different fertilization treatments compared with CK, and the lowest concentration of Cd^2+^ was 1.49 ± 0.196 mg·kg^−1^. The Cd content in the CK group was 3.46 ± 0.174 mg·kg^−1^. The CMP100 treatment group was 56.94% lower than the CK treatment group. Compared with the CK group, Cd^2+^ enrichment in the conventional fertilization treatment group was lower. The enrichment decreased by 15.61–56.94%. The higher the amount of applied Ca^2+^, the higher the concentration of Ca^2+^ in *Brassia campestris* L. and the lower the concentration of Cd^2+^, which indicated that the competitive inhibition of Ca^2+^ and Cd^2+^ could significantly reduce Cd^2+^ absorption by *Brassia campestris* L. roots when absorbing mineral elements (*p* < 0.01).

### 3.5. Effects of Calcium Polypeptide on Available Cadmium Content in Cadmium Contaminated Soil 

**Figure 5:**
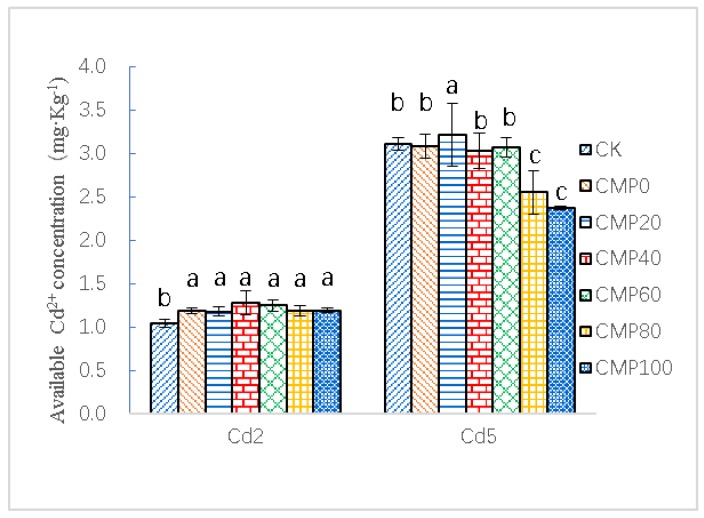
Control of available cadmium in moderately and mildly cadmium-contaminated soils with different amounts of calcium fertilizer. Cd2 and Cd5 indicate that 2.0 and 5.0 mg·Kg^−1^ cadmium-contaminated soil samples were used as the test group; CK stands for control group; CMP0, 20, 40, 60 and 100 stand for treatment groups of urea and calcium polypeptide, according to the same amount of nitrogen and different proportions of calcium application. Different lowercase letters such as a, b, c, d, e, etc. represent significant differences (p < 0.05) between different treatment groups.

As shown in Figure 5, in the contaminated soils containing 2.0 and 5.0 mg·kg^−1^ of cadmium, the content of available cadmium in the soils of each treatment group increased after transplanting. In moderately polluted cadmium soils, effective cadmium could not be passivated, only activated. In heavily polluted cadmium soils, the application of calcium polypeptide reduced the available content of cadmium in soils.

In the contaminated soil containing 2.0 mg·kg^−1^ of cadmium, the available cadmium content of soil in different fertilization treatments increased compared with that of CK. The available cadmium content of soil in the CK group was 1.04 ± 0.042 mg·kg^−1^. The available cadmium soil content in CMP0 was 1.19 ± 0.037 mg·kg^−1^, 13.67% higher than that of CK. Compared with CK, the available cadmium content of calcium polypeptide treatment groups had all increased by 13.22–22.97%.

In the 5.0 mg·kg^−1^ contaminated soil, except for the CMP20 treatment, CK increased by 3.43% compared with the CK control group, and the other fertilization treatments decreased. Compared with CK, CMP40 (3.027 ± 0.201 mg·kg^−1^) had the best effect in reducing the available cadmium content in soil (25.12%; *p* < 0.01).

## 4. Discussion

### 4.1. Correlation Analysis

In the experimental soil with 2.0 and 5.0 mg·kg^−1^ cadmium, six different calcium application gradients were set to apply calcium polypeptide, and the same nitrogen application amount was supplemented with urea according to the optimum nitrogen application amount of *Brassia campestris* L., 0.33 g·kg^−1^. After 40 days, the correlation between the fresh weight of *Brassia campestris* L., the content of *chlorophyll a*, Ca^2+^, and Cd^2+^, and the content of soil-available cadmium was analyzed. Results are shown in Table 2.

As shown in Table 2, in the polluted soil with 2.0 mg·kg^−1^ cadmium, the fresh weight and *chlorophyll a* content of *Brassia campestris* L. had a significant positive correlation with the calcium content (*r* = 0.875, *p* < 0.01; *r* = 0.772, *p* < 0.01) and a significant negative correlation with the cadmium content of *Brassia campestris* L. (*r* = −0.755, *p* < 0.01; *r* = −0.803, *p* < 0.01), while the calcium content of *Brassia campestris* L. had a significant negative correlation with the cadmium content (*r* = −0.803, *p* < 0.01; *r* = 0.734, *p* < 0.01). There was a significant positive correlation between the soil-available cadmium content and calcium content (*r* = 0.549, *p* < 0.01), but the correlation was not closely related. The fresh weight and *chlorophyll a* content of *Brassia campestris* L. also showed a significant positive correlation with soil-available cadmium, and the correlation was not close (*r* = 0.539, *p* < 0.05; *r* = 0.495, *p* < 0.05). Results showed that the application of water-soluble calcium polypeptide could significantly promote the growth of *Brassia campestris* L., increase its fresh weight and *chlorophyll a* content, turn its leaves green, and improve its quality in medium-concentration cadmium-polluted soil; in the polluted soil with 2.0 mg·kg^−1^ cadmium, the heavy-metal cadmium would have a toxic effect on the production of *Brassia campestris* L. [11,15], and reduce its growth to a certain degree. Degree inhibition, and increases in fresh weight and *chlorophyll a*, were slightly slowed down, but it was still within the stress-concentration range that *Brassia campestris* L. could bear, and the plant could continue to grow. Its roots released organic acids to the rhizosphere soil [54], resulting in a slight increase in the available cadmium in the soil. At the same time, calcium application promoted calcium absorption by *Brassia campestris* L., thus reducing cadmium toxicity [21]; therefore, there was significant positive correlation between the calcium content increase and the growth parameters of *Brassia campestris* L.

In the polluted soil containing 5.0 mg·kg^−1^ cadmium, the fresh weight and *chlorophyll a* content of *Brassia campestris* L. had a significant positive correlation with the calcium content (*r* = 0.828, *p* < 0.01; *r* = 0.866, *p* < 0.01) and a significant negative correlation with the cadmium content of *Brassia campestris* L. (*r* = −0.611, *p* < 0.01; *r* = −0.697, *p* < 0.01), while the calcium content of *Brassia campestris* L. had significant negative correlation with the cadmium content (*r* = −0.673, *p* < 0.01). There was a negative correlation between the soil-available cadmium and calcium content (*r* = −0.174, *p* > 0.05), but it was not significant. The fresh weight and *chlorophyll a* content of *Brassia campestris* L. also showed a negative correlation with soil-available cadmium, and it was not close (*r* = −0.129, *p* > 0.05; *r* = −0.324, *p* > 0.05). The results showed that the toxic effect of cadmium on *Brassia campestris* L. was enhanced in the polluted soil containing 5.0 mg·kg^−1^ cadmium, and its growth was obviously inhibited to some extent; the increase in fresh weight and *chlorophyll a* were slightly lower than that of the low cadmium concentration [55]. The application of calcium polypeptide promoted the absorption of calcium in *Brassia campestris* L., thus alleviating the cadmium toxicity to *Brassia campestris* L. [25]. The calcium content in *Brassia campestris* L. had a significant positive correlation with the fresh weight, *chlorophyll a*, and other parameters, indicating that the application of calcium polypeptide could alleviate cadmium toxicity and significantly promote the growth of *Brassia campestris* L. [56].

### 4.2. Effects of Different Calcium Application Rates on Ca^2+^ Accumulation in Brassia campestris L. under Cadmium Stress

Nitrogen (N), phosphorus (P), potassium (K) and calcium (Ca) are essential elements for the growth of *Brassia campestris* L. However, between them, Ca^2+^ plays a very special role [28]. It acts not only as a structural substance of cells, but also as a secondary messenger to regulate the response process of plants to environmental changes. Under adverse conditions, the absorption and distribution of mineral elements in plants also change [26]. Calcium (Ca^2+^) can maintain the integrity of plant-cell membranes and resist stress damage. When the environment changes, e.g., because of heavy-metal stress and elevated salt concentration, the activity of free Ca^2+^ in the cytoplasm increases, which changes protein kinase activity in vivo and induces the expression of related genes. In addition, by regulating the activity of H^+^-ATPase, Ca^2+^ can play the role of stress-tolerance regulation in plants [33]. Therefore, Ca^2+^ uptake is closely related to the distribution and absorption of heavy-metal ions in cells. Increasing the supply level of exogenous Ca^2+^ can reduce the uptake of Cd^2+^ and Pb^2+^ by plants or alleviate the toxicity of heavy metals. Under normal fertility conditions, the supply of Ca^2+^ in soil should far exceed the normal growth requirement of plants, and Ca^2+^ enrichment is common in rhizosphere soil. However, under the condition of insufficient supply of Ca^2+^ in soil and adverse factors affecting Cd^2+^ uptake, such as heavy metal Cd^2+^ stress, Ca^2+^ deficiency seriously affects Ca^2+^ uptake. 

Results have shown that plant biomass increases with the increase in the Ca^2+^ level within 5 mmol·L^−1^ of the Ca^2+^ concentration [26]. This is consistent with the results of this experiment. After 40 days of calcium polypeptide application, the maximum fresh weight of a single plant of *Brassia campestris* L. in two Cd-polluted soils with different concentrations was 34.63 ± 3.54 and 32.89 ± 4.53 g, respectively, which were 3.09% (*p* < 0.05) and 7.95% (*p* < 0.05) lower than that of CMP100 (35.73 ± 3.54 g) in normal soils under the same conditions. However, with the decrease of calcium application, the difference in biomass was gradually increased. Therefore, the application of calcium polypeptide is of great significance to prevent the excessive absorption and toxicity of heavy metals [57,58].

As the ion channels of Ca^2+^ can pass through different cations, specificity is not strong, which is also an important physiological basis for plants to absorb nonessential elements, and for the interaction between Ca^2+^ and other cations [20]. Under the condition of exogenous application of calcium polypeptide, the N and Ca^2+^ provided in this experiment significantly promote the growth of *Brassia campestris* L. and increase the biomass of a single plant, which inevitably leads to *Brassia campestris* L. absorbing more N, P, K, and other nutrients [59]. It is generally believed that K^+^ and Ca^2+^ are antagonistic in absorption. When K^+^ does not exist, it is beneficial to the absorption of Ca by plants. When plants absorb a large amount of Ca^2+^, it hinders the absorption of K^+^. In fact, the relationship between K^+^ and Ca^2+^ is relatively complex. On the one hand, Ca^2+^ is conducive to maintaining cell integrity and the permeability of membrane pores, preventing the leakage of K^+^ in cells, and facilitating K^+^ absorption; on the other hand, when Ca^2+^ reaches a certain concentration, it can compete with K^+^ for absorption sites on the plasma membrane, showing an obvious antagonistic effect [60]. Results have shown that, with the increase in calcium application, the K^+^ content of *Brassia campestris* L. gradually increased, showing a significant synergistic effect. When the K^+^ content reached a certain value, the antagonistic effect was dominant and showed a gradual downward trend, which was completely consistent with the above mechanism.

### 4.3. Control of Calcium Polypeptide on Cadmium Existence in Soil

Calcium polypeptide was obtained by mixing quicklime with plant meal as raw material and reacting for 1–6 h at 130 °C. The content of quicklime was 10–45% (*w/w*) of protein waste. Under high temperatures, the protein components in the plant meal were converted into water-soluble calcium polypeptide. The pH of the aqueous solution ranged from 8.5 to 11.0. In low Cd 2.0 mg·kg^−1^ contaminated soil, by applying the same amount of nitrogen and different proportions of calcium fertilizer combination, cadmium in the soil was slightly activated after 40 days, and the effective concentration of cadmium increased by 0.14–0.23 mg·kg^−1^ compared with the CK control because the effect of cadmium in low Cd^2+^ contaminated soil on *Brassia campestris* L. was not enough to cause toxicity [19,30]. On the contrary, cadmium in low Cd^2+^ contaminated soil can promote plant growth, especially in the case of calcium deficiency. However, the root-tip cells were still affected by Cd^2+^ stress, and their response to stress was reflected in the secretion of organic acids by the roots of *Brassia campestris* L., which lowered the pH of rhizosphere soil. Many studies have shown that soil pH is negatively correlated with the available cadmium in soil. The forms of cadmium were transformed into free and effective forms [61]. In the soil polluted with 5.0 mg·kg^−1^ Cd^2+^, the increase in the Cd^2+^ content led to the increase of toxicity to *Brassia campestris* L. Growth of *Brassia campestris* L. was reduced, the ability of root exudation of organic acids was weakened, and the alkalinity of calcium polypeptide itself increased soil pH and Cd in the soil. The exchange state, mainly in the form of the binding state, and cadmium bioavailability were decreased.

### 4.4. Competitive Inhibitory Effect of Calcium polyPeptide on Cadmium Absorption in Brassia campestris L.

Ca^2+^, a necessary element for plant growth, plays an important role in plant growth, development, and signal transduction. Cd^2+^ has the same valence as Ca^2+^, and the ion radius is extremely close (Cd^2+^ is 9.7 × 10^−2^ nm and Ca^2+^ is 9.9 × 10^−2^ nm). They compete for cation exchange sites on clay minerals, oxides, and organic matter in soil. Ca^2+^ can be replaced by toxic Cd^2+^, and Cd^2+^ can be transported through Ca^2+^ channels [62,63]. The presence of Cd^2+^ may change the level of Ca^2+^ in plant cells, thus affecting normal plant growth and metabolism [63]. Adding the appropriate amount of Ca^2+^ to soil alleviates cadmium toxicity [57,64]. As shown in Figure 4, results showed that in the contaminated soils containing 2.0 mg·kg^−1^ cadmium, when the same amount of nitrogen and different proportion of calcium fertilizer were applied, cadmium in the soil was slightly activated after 40 days, and effective cadmium concentration increased by 0.14–0.23 mg·kg^−1^ when compared with the CK control. The secretion of organic acids by the roots of *Brassia campestris* L. lowered the pH of rhizosphere soil [54]. Cadmium forms were transformed into free and effective forms. However, the Ca^2+^ enrichment of *Brassia campestris* L. significantly increased, and the highest Ca^2+^ enrichment of *Brassia campestris* L. was 34.61 ± 3.28 mg·g^−1^ (DW) when the application of Ca^2+^ reached the maximum, which was 78.42% higher than that of CK, with a significant difference (*p* < 0.01). After 40 days of growth, there was a significant negative linear relationship between Cd^2+^ concentration and calcium application (*r* = −0.9822), and the lowest Cd^2+^ concentration of *Brassia campestris* L. was 0.84 ± 0.003 mg·kg^−1^, which was 64.71% lower than that of the CK control (*p* < 0.01).

Similarly, in 5.0 mg·kg^−1^ cadmium-contaminated soil, the available cadmium content of all treatments except for CMP20 increased by 3.43% compared with that of CK, while those of the other fertilization treatments decreased. The effective cadmium content of CMP40 (3.027 ± 0.201 mg·kg^−1^) was reduced by 25.12% compared with that of CK (*p* < 0.01), which showed a significant difference between them. This is consistent with when calcium polypeptide was produced in an alkaline condition and was weakly alkaline. It is equivalent to the increase in soil pH after the application of calcium polypeptide, which is much larger than the range of organic acids secreted by roots. Therefore, the available cadmium in soil was significantly decreased. At this time, the concentration of Ca^2+^ in *Brassia campestris* L. significantly increased, and the highest concentration was 34.52 ± 2.57 mg·g^−1^ (DW), 44.90% higher than that of CK (*p* < 0.01). Otherwise, the relationship between calcium content and calcium application was linear (*r* = 0.727). After 40 days of growth, there was significant negative correlation between the Cd^2+^ concentration and Ca^2+^ application (*r* = −0.9886). The lowest concentration of Cd^2+^ in *Brassia campestris* L. was 0.84 ± 0.003 mg·kg^−1^, 64.71% lower than that of CK (*p* < 0.01). This showed that, in the two Cd^2+^ polluted soils, Ca^2+^ and Cd^2+^ had obvious competitive inhibition. With the increase in calcium polypeptide application, Cd^2+^ did not significantly inhibit the absorption or transport of Ca^2+^ by *Brassia campestris* L., and *Brassia campestris* L. had obvious Ca^2+^ enrichment. Similarly, due to the competition and regulation effect of Ca^2+^, the absorption of Cd^2+^ by *Brassia campestris* L. decreased, which is similar to the toxic effect of Ca^2+^ on plants [27].

## 5. Conclusions

In this study, six different calcium application gradients were used to apply calcium polypeptides to the soil with cadmium contamination. The competitive inhibition of calcium polypeptides application on Cd^2+^ uptake by *Brassia campestris* L. and its growth processes under cadmium toxicity were discussed. The results showed that the application of calcium polypeptides significantly promotes the growth of *Brassia campestris* L., and the maximum fresh weight of a single plant could be increased by 27.53% (*p* < 0.05) and 12.84% (*p* < 0.05), respectively, while the *chlorophyll a* content accordingly increased by 5.72% (*p* > 0.05) and 0.61% (*p* > 0.05), compared with that of urea alone. The application of calcium polypeptide could also alleviate the effect of Cd^2+^ stress on the growth of *Brassia campestris* L., promoting the absorption of K^+^, Ca^2+^ and other nutrient ions by *Brassia campestris* L. The inhibition of calcium polypeptides on cadmium uptake by *Brassia campestris* L. is mainly through the competitive inhibition of calcium on cadmium rather than through a passivation effect. Under different calcium polypeptides application conditions, the effective state of Cd^2+^ in soil was not significantly reduced, which indicated that calcium polypeptide had limited or weak passivation effect on Cd^2+^. By applying calcium polypeptides, however, Cd^2+^ enrichment and calcium polypeptides application of *Brassia campestris* L. were significantly increased. It showed that the calcium polypeptides could not only promote the growth of *Brassia campestris* L. but also had an obvious competitive inhibition effect on the enrichment of heavy-metal Cd^2+^ in plants, having the dual functions of a fertilizer and competitive inhibition of heavy metals. This study provides a new technology for the in-situ remediation of heavy-metal pollution for the treatment of Cd^2+^-contaminated farmland and surrounding land.

## Figures and Tables

**Table 1:** Basic physical and chemical properties of experimental soils.

Organic Matter (g·kg^−1^)	Total Nitrogen (g·kg^−1^)	Total Phosphorus (g·kg^−1^)	Total Potassium (g·kg^−1^)	Alkali-Hydrolyzed Nitrogen (mg·kg^−1^)	Total Cadmium (mg·kg^−1^)	pH
13.50	1.437	0.735	8.53	98.03	0.002	7.1

**Table 2:** Pairwise Pearson correlation coefficients among various indicators for cadmium-contaminated soil (*n* = 21). Cd5 is below the diagonal, and Cd2 is above the diagonal.

Indicators	Fresh Weight	*Chlorophyll a* Content	Calcium Content	Cadmium Content	Available Cd^2+^ Concentration
Fresh weight	1	0.772 **	0.875 **	−0.755 **	0.539 *
*Chlorophyll a* content	0.734 **	1	0.756 **	−0.803 **	0.495 *
Calcium content	0.828 **	0.866 **	1	−0.734 **	0.594 **
Cadmium content	−0.611 **	−0.697 **	−0.673 **	1	−0.530 *
Available Cd^2+^ concentration	−0.129	−0.324	−0.174	0.442*	1

* Significant correlations at the 0.05 level (*p* < 0.05, both sides); ** significant correlation at the 0.01 level (*p* < 0.01, both sides).
